# Quality of healthcare services to reduce leprosy in Brazil: a trend analysis from 2001 to 2020

**DOI:** 10.1590/1980-549720240034

**Published:** 2024-07-01

**Authors:** Gabriel da Silva Mártires, Géssica Liana dos Santos Lima, Danilo Esteves Gomes, Angelina do Carmo Lessa, Celsa da Silva Moura Souza, Eliane Ignotti, Ronilson Ferreira Freitas

**Affiliations:** I Universidade Federal do Amazonas, School of Medicine – Manaus (AM), Brazil.; II Universidade Federal dos Vales do Jequitinhonha e Mucuri, Postgraduate Program in Health, Society and Environment – Diamantina (MG), Brasil.; III Universidade Federal do Amazonas, Postgraduate Program in Health Sciences – Manaus (AM), Brazil.; IV Universidade do Estado do Mato Grosso, Postgraduate Program in Environmental Sciences – Cáceres (MT), Brazil.; V Universidade Federal do Amazonas, Postgraduate Program in Family Health – Manaus (AM), Brazil.

**Keywords:** Epidemiology, Temporal distribution, Leprosy, Health status indicators, Public health

## Abstract

**Objective:**

To analyze the temporal trend of healthcare services quality indicators to reduce leprosy in Brazil, over a 20-year period.

**Methods:**

This is an epidemiological study with a temporal trend, whose data were extracted from the Notifiable Diseases Information System. Indicators were constructed from the Ministry of Health Technical-Operational Manual that presents the Guidelines for Surveillance, Care and Elimination of Leprosy as a Public Health Problem. For trend analysis of the selected indicators, the Prais-Winsten model was used and the Average Annual Growth Rate (AAGR) was also calculated.

**Results:**

In the 20-year time series investigated here, 732,959 cases of leprosy were reported in Brazil. The trend was stationary for: new leprosy cases cure rate (β=-0.000; p=0.196; AAGR=-0.2), new leprosy cases drop out rate (β=-0.001; p=0.147; AAGR=-0.4), new leprosy cases contact tracing rate (β=-0.001; p=0.112; AAGR=1.6), new cases of leprosy with degree physical disability assessment rate among new cases (β=-0.000; p=0.196; AAGR=-0.2) and cases cured in the year with the degree of physical disability assessed (β=0.002; p=0.265; AAGR=0.5); while the indicator of recurrence rate among cases reported in the year (β=0.019; p<0.001; AAGR=0.5) showed an increasing trend.

**Conclusion:**

Based on the evaluation of indicators to assess the quality of healthcare services to reduce leprosy, it was evident that Brazil has major challenges for its full implementation, with improvements being necessary in the quality of care service offered to the population.

## INTRODUCTION

Leprosy remains a significant public health challenge due to its widespread prevalence and considerable disability impact, particularly affecting vulnerable and marginalized populations in middle and low-income countries^1^. Despite the implementation of policies and programs by the Ministry of Health aimed at its eradication^2^, leprosy continues to exhibit a high prevalence in Brazil^3^ — which, globally, ranks second in number of reported cases — being classified, therefore, as a neglected disease with the potential for elimination^3^.

In 2020, a total of 127,396 new cases of leprosy were reported worldwide. Within the American continent, 19,195 (15.1%) cases were registered; of these, 17,979 were reported in Brazil, corresponding to 93.6% of the new cases reported in the Americas^4^.

In the 1980s, the World Health Organization (WHO) introduced polychemotherapy (poly-CT) as the treatment protocol for leprosy, a measure aimed at halting transmission and preventing deformities, which was also adopted in Brazil^5^. Subsequently, in 2002, the Ministry of Health established operational indicators to evaluate the effectiveness of interventions and services aimed at reducing and monitoring leprosy^6^. In 2016, with the publication of the Guidelines for Surveillance, Care, and Elimination of Leprosy as a Public Health Problem, operational indicators were designed to assess the quality of healthcare services in reducing leprosy^7^.

While literature offers studies on trend analysis of leprosy indicators, they predominantly concentrate on local and/or regional contexts, lacking publications with national coverage^8-10^. Furthermore, these studies often assess indicators over short time intervals^9,11^. Another notable gap is the limited research evaluating the quality of healthcare services aimed at reducing leprosy, as the majority of studies have focused on epidemiological indicators^11-13^.

Therefore, studies employing indicators to assess the quality of healthcare services aimed at reducing leprosy at a national level enable the monitoring of the impact of already implemented public policies. They also offer support to health managers in planning, decision-making, and program execution or improvements addressing the issue of leprosy in Brazil. Consequently, this study aimed to analyze the temporal trend of quality indicators of healthcare services to reduce leprosy in Brazil over a 20-year period.

## METHODS

This epidemiological study examined temporal trends using indicators to assess the quality of healthcare services aimed at reducing leprosy in Brazil, encompassing a historical series from 2001 to 2020.

The data were sourced from the Notifiable Diseases Information System (*Sistema de Informação de Agravos de Notificação* – SINAN) of the Ministry of Health, managed by the Information Technology Department of the Unified Health System (*Departamento de Informática do Sistema Único de Saúde* – DATASUS)^14^. Notifications with code A-30 were specifically chosen, falling under the “leprosy” category, recorded within the selected timeframe, and classified according to the criteria of the International Statistical Classification of Diseases and Related Health Problems (Tenth Revision), ICD-10^15^.

To assess the quality of healthcare services aimed at reducing leprosy, six indicators were employed. These indicators were derived from the Technical-Operational Manual, which outlines the Guidelines for Surveillance, Care, and Elimination of Leprosy as a Public Health Problem, issued by the Ministry of Health^7^ (Chart 1).

**Chart 1:** Indicators used to evaluate the quality of health care services for reducing leprosy in Brazil^7^.

Indicator	Construction	Utility	Parameters
Proportion of leprosy cure among newly diagnosed cases in the cohort years.	Numerator: New leprosy cases residing in a specific location, diagnosed in the cohort years, and cured by December 31st of the evaluation year. Denominator: Total new leprosy cases residing in the same location and diagnosed in the cohort years. Multiplication factor: 100.	Evaluate the quality of care and follow-up of new cases diagnosed until treatment completion.	Good: =90%; Regular: =75 to 89.9%; Poor: <75%
Proportion of leprosy cases in treatment abandonment among newly diagnosed cases in the cohort years.	Numerator: New leprosy cases diagnosed in the cohort years who abandoned treatment by December 31^st^ of the evaluation year. Denominator: Total new leprosy cases diagnosed in the cohort years. Multiplication factor: 100.	Assess the quality of care and monitoring of newly diagnosed cases until treatment completion.	Good: <10%; Regular: 10 to 24.9%; Poor: =25%.
Proportion of contacts examined among newly diagnosed leprosy cases in the cohort years.	Numerator: Number of contacts of new leprosy cases examined by current residence location and diagnosed in the cohort years (PB diagnosed in the year before the evaluation year and MB diagnosed two years before the evaluation year). Denominator: Total contacts of new leprosy cases recorded by current residence location and diagnosed in the cohort years (PB diagnosed in the year before the evaluation year and MB diagnosed two years before the evaluation year). Multiplication factor: 100.	Measures the capacity of services to conduct surveillance of contacts of newly diagnosed leprosy cases, thereby enhancing the timely detection of new cases.	Good: =90,0%; Regular: =75.0 to 89.9%; Poor: <75.0%.
Proportion of relapse cases among the cases reported in the year.	Number of reported cases of leprosy relapse/total reported cases in the year x 100.	Identify notifying municipalities of relapse cases for monitoring of therapeutic failure.	No parameter specified.
Proportion of new leprosy cases with the degree of physical disability assessed at diagnosis.	Numerator: new cases of leprosy with the degree of physical disability assessed at diagnosis, residing in a specific location and detected in the year of assessment. Denominator: new cases of leprosy, residing in the same location and diagnosed in the year of assessment. Multiplication factor: 100.	Measure the quality of care in health services.	Good: =90%; Regular: =75 to 89.9%; Poor: <75%.
Proportion of cases cured within the year with the degree of physical disability assessed among new leprosy cases in the cohort period.	Numerator: cases cured within the year with the degree of physical disability assessed at the time of cure, residing in a specific location. Denominator: total cases cured within the year residing in the same location. Multiplication factor: 100.	Measure the quality of care in healthcare services.	Good: =90%; Regular: =75 a 89,9%; Poor: <75%.

The database was constructed using Microsoft Excel software, facilitating the calculation of proportions. Statistical analyses to assess the temporal trend and AAGR were conducted using the Stata statistical package (version 13.0).

To analyze the trend of indicators for assessing the quality of healthcare services aimed at reducing leprosy in Brazil, the Prais-Winsten linear regression test was employed^16^. The proportions corresponding to each evaluated indicator were considered as dependent variables, with the independent variable being the years within the historical series.

In the trend analysis, the β value of the proportions for each evaluated indicator was derived, representing the slope of the straight line. The rate of variation was utilized to classify the trend as follows: a positive rate of variation indicates an increasing time series, a negative rate of variation indicates a decreasing trend, and the trend is considered stationary when there is no significant difference between its value and zero. The level of significance was determined by comparing the p-value with the value provided by the standard normal curve, with a 95% Confidence Interval (CI). For all indicators, those with a model estimate yielding a p-value of <0.05 were deemed significant^17^.

The quantitative estimate of the trend is calculated by the following equation:

$$\text{AAGR=}\left({\text{-1+10}}^{\text{β}}\right)\text{*100}$$

Where:

β corresponds to the angular coefficient formed in the linear regression.

To calculate the CI of the study measurements, the following formula was used:

$$\text{95\%CI=}\left(\text{-1+10}\left(\text{β}\pm \text{r*EP}\right)\right)\text{*100}$$

Where:

t = value at which Student’s *t* distribution has 19 degrees of freedom at a two-tailed 95% CI;

SE = standard error of the estimate of β, provided by the regression analysis.

For all statistical tests performed, a significance level of 5% was adopted. Therefore, values of p≤0.05 were considered significant.

Following the criteria established by Resolution No. 466/2012^18^ and Resolution No. 510/2016^19^ of the National Health Council for research involving human subjects, studies that utilize publicly accessible information, as per the terms of Law No. 12.527^20^, dated November 18, 2011, are not required to be registered or evaluated by the Research Ethics Committee (*Comitês de Ética em Pesquisa* – CEP)/National Commission for Ethics in Research (*Comissão Nacional de Ética em Pesquisa* – CONEP).

## RESULTS

In the 20-year time series under investigation, a total of 732,959 cases of leprosy were reported in Brazil. Within this period, 545,610 cases of cure were documented. The overall proportion of leprosy cure among new cases diagnosed throughout the cohort years from 2001 to 2020 was 84.49%. The lowest proportion of cure was observed in 2019, while the highest was in 2007. Regarding the indicator for evaluating service quality, all years in the series — except for 2019, which was classified as precarious — were labeled as regular. This suggests that despite efforts to control and treat leprosy, effectiveness remains incomplete in Brazil (Table 1).

**Table 1:** Quality of healthcare services for reduction in Brazil, from 2001 to 2020.

Year	Indicators					
	Proportion of cure of leprosy among new cases diagnosed in the cohort years		Proportion of leprosy cases in treatment abandonment among new cases diagnosed in the cohort years		Proportion of contacts examined among new leprosy cases diagnosed in the cohort years.	
	Proportion	Classification	Proportion	Classification	Proportion	Classification
2001	86.97	Regular	6.97	Good	62.03	Poor
2002	87.23	Regular	5.79	Good	52.10	Poor
2003	86.05	Regular	5.67	Good	44.67	Poor
2004	86.56	Regular	5.23	Good	47.21	Poor
2005	87.16	Regular	4.83	Good	53.93	Poor
2006	86.26	Regular	5.24	Good	59.77	Poor
2007	87.47	Regular	5.48	Good	70.14	Poor
2008	87.18	Regular	5.03	Good	68.86	Poor
2009	85.36	Regular	4.92	Good	70.74	Poor
2010	83.72	Regular	4.34	Good	73.02	Poor
2011	84.44	Regular	4.25	Good	75.12	Regular
2012	82.77	Regular	4.51	Good	77.08	Regular
2013	85.16	Regular	4.86	Good	78.93	Regular
2014	83.76	Regular	5.33	Good	79.26	Regular
2015	81.68	Regular	6.04	Good	78.80	Regular
2016	80.74	Regular	6.16	Good	81.01	Regular
2017	79.09	Regular	6.12	Good	82.37	Regular
2018	80.70	Regular	6.13	Good	82.35	Regular
2019	74.68	Poor	7.64	Good	78.87	Regular
2020	77.07	Regular	6.43	Good	73.17	Poor

Regarding treatment abandonment, among new cases diagnosed in the cohort years, 34,999 cases were registered. The proportion of abandonment over the 20-year time series was 5.42%. The year with the highest number of abandonments was 2019, recording 1,971 cases, while the lowest number was in 2011 (1,425). In terms of the health care service quality parameter for the treatment abandonment indicator, all years received a good rating. During the investigated period, 2,131,711 contacts of new leprosy cases diagnosed in the cohort years were recorded, with 1,439,380 examined, representing a proportion of 67.5%. From 2001 to 2010, the quality of health service regarding the proportion of examined contacts of new leprosy cases diagnosed in the cohort years was classified as precarious. From 2011 to 2020, they were classified as regular, indicating the need to enhance health surveillance services (Table 1).

Over the span of 20 years, 30,873 cases of recurrence were recorded, constituting a proportion of 4.1 among the cases reported during this period. The highest proportion of relapse occurred in 2018, while the lowest was observed in 2001. Regarding the proportion of new leprosy cases with a degree of physical disability assessed at diagnosis, an average of 88.9% was noted from 2001 to 2020. During this period, only the years 2006, 2009, 2010, and 2011 received a good classification for this indicator. The remaining years were classified as regular (Table 2).

**Table 2:** Quality of health care services for leprosy reduction in Brazil, from 2001 to 2020.

Year	Indicators					
	Proportion of relapse cases among cases reported in the year*		Proportion of new leprosy cases with assessed degree of physical disability at diagnosis		Proportion of cases cured in the year with assessed degree of physical disability among new leprosy cases in the cohort period	
	Proportion	Classification	Proportion	Classification	Proportion	Classification
2001	2.7	-	88.29	Regular	62.96	Poor
2002	2.8	-	88.09	Regular	61.33	Poor
2003	2.9	-	88.70	Regular	60.77	Poor
2004	3.1	-	88.66	Regular	60.44	Poor
2005	3.4	-	89.38	Regular	60.67	Poor
2006	3.5	-	90.70	Good	59.22	Poor
2007	3.5	-	89.15	Regular	70.18	Poor
2008	3.8	-	89.33	Regular	73.36	Poor
2009	3.9	-	90.33	Good	73.88	Poor
2010	3.9	-	90.69	Good	73.75	Poor
2011	4.3	-	90.74	Good	72.25	Poor
2012	4.7	-	89.87	Regular	71.62	Poor
2013	4.6	-	89.47	Regular	71.42	Poor
2014	4.8	-	88.77	Regular	70.30	Poor
2015	5.3	-	88.73	Regular	69.59	Poor
2016	5.6	-	88.64	Regular	69.03	Poor
2017	6.0	-	88.58	Regular	68.45	Poor
2018	6.3	-	88.15	Regular	70.39	Poor
2019	5.7	-	87.29	Regular	68.21	Poor
2020	5.8	-	84.60	Regular	67.81	Poor

* No classifcation parameter specified.

Regarding the proportion of cases cured in the year with the degree of physical disability assessed among new cases of leprosy in the cohort period, the average proportion observed between 2001 and 2019 was 67.8%. This result was considered precarious (Table 2).

In the temporal trend analysis of health service quality indicators to reduce leprosy in Brazil, a stationary trend was observed for the following indicators: proportion of leprosy cure among newly diagnosed cases (AAGR=-0.2; 95%CI=-0.5-0.1); proportion of leprosy cases that abandon treatment among new cases diagnosed (AAGR=-0.4; 95%CI=-1.5-0.2); proportion of examined contacts of newly diagnosed leprosy cases (AAGR=1.6; 95%CI=-0.4-3.6); proportion of new leprosy cases with degree of physical disability assessed at diagnosis (AAGR=-0.2; 95%CI=-0.5-0.1); and proportion of cured cases in the year with the degree of physical disability assessed among new leprosy cases in the cohort period (AAGR=0.5; 95%CI=-0.4-1.5). However, the indicator of proportion of recurrence cases among cases reported in the year (AAGR=0.5; 95%CI=-0.4-1.5) showed an increasing trend (Table 3).

**Table 3:** Temporal trend of indicators assessing the quality of health care services for leprosy reduction in Brazil from 2001 to 2020.

Indicators	Prais-Winsten					Average Annual Growth Rate (AAGR%)			Trend
	ß	95%CI		p value*	R^2^	TIA%	95%CI		
		Lower	Upper				Lower	Upper	
Proportion of leprosy cure among new cases diagnosed in the cohort years	-0.000	-0.002	0.000	0.196	0.999	-0.2	-0.5	0.1	Stationary
Proportion of leprosy cases in treatment abandonment among new cases diagnosed in the cohort years	-0.001	-0.004	0.000	0.147	0.967	-0.4	-1.0	0.2	Stationary
Proportion of contacts examined for new leprosy cases diagnosed in the cohort years	0.006	-0.001	0.015	0.112	0.955	1.6	-0.4	3.6	Stationary
Proportion of leprosy relapse cases among those reported in the year	0.019	0.016	0.021	<0.001	0.916	0.5	-0.4	1.5	Growing
Proportion of new leprosy cases with assessed degree of physical disability at diagnosis	-0.000	-0.002	0.000	0.196	0.999	-0.2	-0.5	0.1	Stationary
Proportion of cases cured in the year with assessed degree of physical disability among new leprosy cases in the cohort period.	0.002	-0.001	0.006	0.265	0.987	0.5	-0.4	1.5	Stationary

95%CI: confidence interval; R^2^: coefficient of determination; AAGR: average annual growth rate.

* Significance level p<0.05

## DISCUSSION

The findings of this study highlight the precarious quality of care for individuals diagnosed with leprosy, as well as shortcomings in surveillance capabilities and the effectiveness of actions for early case detection. These findings indicate a deviation from the standards recommended by the WHO^21^.

The trend analysis of the leprosy cure proportion indicator revealed unsatisfactory outcomes for individuals affected by the disease until the completion of treatment and achieving cure. A study conducted in Maranhão between 2002 and 2015, a period similar to that of the data presented here, also found a stationary trend for this indicator, that is, no progress in the number of cured cases^22^. Similarly, Machado^23^, in a historical series from 2003 to 2015, analyzing risk clusters for leprosy in Brazil, observed a stationary trend for the cure rate in most clusters, specifically in 14 out of the 15 analyzed. This underscores the importance of involving health professionals in the treatment adherence process and the necessity for health education programs to enhance individuals’ knowledge about leprosy. Improving the proportion of cured cases can facilitate the interruption of transmission and reduce instances of disability^23,24^.

The temporal trend of the indicator proportion of leprosy cases abandoning treatment was investigated, as it is inversely proportional to the effectiveness of the leprosy control program^25^, and it showed a stationary trend. A study conducted in Brazil between 2001 and 2015 reported an increase in the proportion of leprosy treatment abandonment^26^. These findings highlight the low effectiveness of the strategy to reduce treatment abandonment^27^, which could result in subtherapeutic dosing, leading to drug resistance and treatment failure^25,28^.

The indicator proportion of examined contacts of newly diagnosed leprosy cases exhibited a stationary trend, indicating that the capacity of services to conduct surveillance of new leprosy cases and promptly detect them is still considered precarious. Despite an improvement in classification during the second decade, transitioning from precarious to regular, the onset of the COVID-19 pandemic in 2020 likely caused a decline in the proportion, resulting in the indicator being classified as precarious once again. In a historical series presented by Souza et al.^29^, with data from the state of Bahia spanning from 2003 to 2014, an increasing trend was observed; however, even with this trend, the indicator was still classified as precarious. The approach to contacts is crucial for disease control, as it can provide counseling and systematic long-term monitoring of individuals and families at risk of illness, considering the disease’s spread characteristics^29^.

Regarding the indicator proportion of new leprosy cases with degree of physical disability assessed at diagnosis, this indicator exhibited a stationary trend, maintaining a regular pattern throughout all years of the series. In the state of Paraíba, this indicator also demonstrated a stationary trend from 2001 to 2014; however, its classification remained precarious throughout the series, not exceeding 59%^30^. Conversely, in Minas Gerais, a state in the region with the lowest occurrence of the disease, although the indicator was classified as good, exceeding 90%, from 2008 to 2018, its trend was downward^31^. Therefore, it is crucial to note that the trend and classification of this indicator presented in this study, with data for Brazil, conceal the significant variability that exists between regions of the country. In this context, monitoring the implementation of active tracking programs for leprosy cases becomes essential, providing opportunities for disease control and reducing the proportion of cases with a degree of physical disability at the time of diagnosis^32,33^.

The indicator proportion of cases cured in the year with the degree of physical disability assessed among new cases of leprosy is crucial for evaluating the effectiveness of disease control strategies. Early diagnosis and treatment can mitigate the risk of physical disability and consequently improve the quality of life for patients^34^. A stationary trend was observed for this indicator, which, throughout all years of the investigated series, was classified as precarious. In Minas Gerais, a study conducted from 2008 to 2018 reported a regular classification for this indicator, but its decreasing trend led to a precarious level by the end of the series^31^. These findings highlight persistent challenges in evaluating and monitoring post-cure patients to ensure comprehensive care, including assistance for rehabilitation if needed. This aligns with the recommendations of the Brazilian Unified Health System (*Sistema Único de Saúde* – SUS) for continued care and support^11,25,33^.

In the present study, the indicator proportion of recurrence cases among cases reported in the year exhibited an increasing trend. A time series study conducted in Bahia from 2001 to 2014 similarly noted an increasing trend for this indicator, pointing to a rise in the number of municipalities reporting recurrence cases from 2008 to 2014^35^. Various predictive factors contribute to recurrence in patients diagnosed and cured, including treatment failure, reinfection linked to housing conditions and lifestyle habits, and the organization of health services. Additionally, professionals’ failure to differentiate recurrences from reactions post-discharge can also influence recurrence rates^7,36^. Given this perspective and considering the upward trend of the indicator, there is a pressing need to develop and implement strategies aimed at reducing determinants contributing to recurrence cases^37,38^. It is crucial for services to distinguish relapse from situations involving reverse leprosy reaction, therapeutic insufficiency, and therapeutic failure. Cases unresponsive to proposed treatments for reactional states should be referred to reference units to confirm recurrence^39^.

In summary, Brazil still faces significant challenges on the path to eradicating leprosy, as it remains among the five countries that have not achieved the control target proposed by the WHO, persisting with high levels of endemicity^40^. The continual high number of new cases poses a substantial obstacle to leprosy elimination, contributing to the emergence of new cases through household contacts^41,42^. As demonstrated in this study, efforts to conduct contact exams, along with other indicators proposed by the leprosy control strategy, have fallen short. Moreover, the high incidence of the disease is closely intertwined with social determinants. Therefore, endemic countries like Brazil should integrate the eradication of poverty into their health policies. Actions within the health sector alone are deemed insufficient to address the diverse needs of the socially and economically marginalized populations^7,21,43,44^.

Although the results underscore the imperative to enhance the quality of healthcare services to reduce leprosy, it is essential to acknowledge some limitations of this study. The epidemiological design employed restricts the observation and analysis of the quality of leprosy care provided within the specific context of Brazilian municipalities^9^.

The limitations stemming from the utilization of secondary data from DATASUS must also be acknowledged, including gaps in data filling, the potential for underreporting, and inconsistencies in the flow of consolidated data within the system. However, leveraging this data enables access to a national registry encompassing a substantial final population, which is crucial for the epidemiological evaluation of neglected diseases. It can also facilitate assessment by managers and professionals, aiding decision-making and informing public health policy review and actions^17,45^.

Furthermore, the temporal analysis of the effectiveness indicators of preventive measures and the attainment of their goals for eliminating leprosy in Brazil over a 20-year period enables the evaluation of the quality of healthcare services in implementing control actions. This process generates evidence that facilitates the adoption of strategies to address operational control challenges.

Furthermore, it is crucial to conduct further analyses with stratification according to regions so that policies can be tailored to the specific realities of each region of Brazil. Given the heterogeneous prevalence of the disease throughout the country and the vast territorial expanse, as well as the unequal distribution of resources, this adaptation is essential to ensure the effectiveness of health interventions.

Finally, given the current situation of leprosy in Brazil, it is imperative that the commitments outlined by the WHO for the elimination of the disease serve as guiding principles for the actions and policies implemented in the country. Addressing the challenges of overcoming delays in diagnosis, treatment, case monitoring, and active contact tracing requires financial investments to enable adequate training of professionals and improve the quality of healthcare services aimed at reducing leprosy in Brazil.

## References

[1] Brasil. Ministério da Saúde (2017). Guia prático sobre a hanseníase [Internet].

[2] Costa MS, Silva PCB, Moura JPG, Pantoja PVN, Silva MP (2015). Políticas para hanseníase: a evolução da gestão em
saúde. Rev Enf.

[3] World Health Organization (2022). Global leprosy (Hansen disease) update, 2021: moving towards
interruption of transmission [Internet].

[4] World Health Organization (2021). Global leprosy update, 2020: impact of COVID-19 on global leprosy
control. Weekly Epidemiological Record.

[5] Brasil. Ministério da Saúde (2000). Legislação sobre o controle da hanseníase no Brasil [Internet].

[6] Ministério da Saúde. Secretaria de Políticas de Saúde (2002). Guia para o controle da hanseníase [Internet].

[7] Brasil. Ministério da Saúde (2016). Secretaria de Vigilância em Saúde. Departamento de Vigilância das
Doenças Transmissíveis. Diretrizes para vigilância, atenção e eliminação da
Hanseníase como problema de saúde pública: manual técnico-operacional
[Internet].

[8] Souza CDF, Matos TS (2018). Análise de tendência dos indicadores de monitoramento e avaliação
da qualidade dos serviços de hanseníase em município prioritário do Nordeste
brasileiro. Rev Bras Pesqui Saúde.

[9] Lima LV, Pavinati G, Silva IGP, Moura DRO, Gil NLM, Magnabosco GT (2022). Temporal trend, distribution and spatial autocorrelation of
leprosy in Brazil: ecological study, 2011 to 2021. Rev Bras Epidemiol.

[10] Barros ICA, Sousa CCM, Silva NRF, Mascarenhas MDM (2024). Characterization of cases and epidemiological and operational
indicators of leprosy: analysis of time series and spatial distribution,
Piauí state, Brazil, 2007-2021. Epidemiol Serv Saúde.

[11] Anchieta JJS, Costa LMM, Campos LC, Vieira MR, Mota OS, Morais OL (2019). Trend analysis of leprosy indicators in a hyperendemic Brazilian
state, 2001–2015. Rev Saúde Pública.

[12] Souza CDF, Paiva JPS, Leal TC, Urashima GS (2020). Hanseníase no Brasil no século XXI: análise dos indicadores
epidemiológicos e operacionais utilizando regressão por pontos de
inflexão. An Bras Dermatol.

[13] Basso MEM, Andrade RF, Silva RLF (2021). Trend of epidemiological indicators of leprosy in an endemic
state of the Amazon region. Rev Gaucha Enferm.

[14] Brasil. Ministério da Saúde. Sistema de Informação de Agravos de
Notificação Departamento de Informática do Sistema Único de Saúde. Acompanhamento
dos dados de hanseníase – Brasil [Internet].

[15] Organização Mundial da Saúde (1994). Classificação estatística internacional de doenças e problemas
relacionados à saúde – CID-10.

[16] Antunes JLF, Cardoso MRA (2015). Uso da análise de séries temporais em estudos
epidemiológicos. Epidemiol Serv Saúde.

[17] Gonçalves ICM, Freitas RF, Aquino EC, Carneiro JA, Lessa AC (2022). Mortality trend from falls in Brazilian older adults from 2000 to
2019. Rev Bras Epidemiol.

[18] Brasil. Ministério da Saúde. Conselho Nacional de Saúde (2012). Resolução no 466, de 12 de dezembro de 2012. Aprova diretrizes e normas
regulamentadoras de pesquisas envolvendo seres humanos [Internet].

[19] Brasil. Ministério da Saúde (2016). Conselho Nacional de Saúde. Resolução no 510, de 07 de abril de 2016
[Internet].

[20] Brasil. Presidência da República. Casa Civil (2011). Subchefia para Assuntos Jurídicos. Lei no 12.527, de 18 de novembro de
2011. Regula o acesso a informações previsto no inciso XXXIII do art. 5º, no
inciso II do § 3º do art. 37 e no § 2º do art. 216 da Constituição Federal;
altera a Lei no 8.112, de 11 de dezembro de 1990; revoga a Lei no 11.111, de
5 de maio de 2005, e dispositivos da Lei no 8.159, de 8 de janeiro de 1991;
e dá outras providências [Internet].

[21] Organização Mundial da Saúde. Rumo à zero hanseníase (2021). Estratégia global para a hanseníase 2021-2030 [Internet].

[22] Anchieta JJS, Costa LMM, Campos LC, Vieira MR, Mota OS, Morais OL (2019). Trend analysis of leprosy indicators in a hyperendemic Brazilian
state, 2001–2015. Rev Saude Publica.

[23] Machado RNR (2019). Descentralização das ações de controle da hanseníase nos clusters de
risco do Brasil [dissertação de mestrado].

[24] Machado LMG, Santos ES, Cavaliero A, Steinmann P, Ignotti E (2022). Spatio-temporal analysis of leprosy risks in a municipality in
the state of Mato Grosso-Brazilian Amazon: results from the leprosy
post-exposure prophylaxis program in Brazil. Infect Dis Poverty.

[25] Souza EA, Boigny RN, Ferreira AF, Alencar CH, Oliveira MLW, Ramos AN (2018). Vulnerabilidade programática no controle da hanseníase: padrões
na perspectiva de gênero no Estado da Bahia, Brasil. Cad Saúde Pública.

[26] Felix E, Santos VC, Paixão AS, Sampaio KCP, Anjos KF (2018). Perfil epidemiológico da hanseníase no Brasil no período de 2001
a 2015. Revista Brasileira de Saúde Funcional.

[27] Lira KB, Leite JJG, Maia DCBSC, Freitas RMF, Feijão AR (2012). Knowledge of the patients regarding leprosy and adherence to
treatment. Braz J Infect Dis.

[28] Moraes MO (2016). Editorial commentary: Drug-resistance in leprosy: moving toward
understanding the scope of the problem and how to tackle it. Clin Infect Dis.

[29] Souza EA, Ferreira AF, Pinto MSAP, Heukelbach J, Oliveira HX, Barbosa JC (2019). Desempenho da vigilância de contatos de casos de hanseníase: uma
análise espaço-temporal no Estado da Bahia, Região Nordeste do
Brasil. Cad Saúde Pública.

[30] Brito KKG, Andrade SSC, Santana EMF, Peixoto VB, Nogueira JA, Soares MJGO (2015). Epidemiological analysis of leprosy in an endemic state of
northeastern Brazil. Rev Gaúcha Enferm.

[31] Lages DS, Kerr BM, Bueno IC, Niitsuma ENA, Vidal SL, Reis GCS (2022). Avaliação do grau de incapacidade física por hanseníase em Minas Gerais.
In: Farias HPS, org. Educação, saúde e sociedade: investigações, desafios e
perspectivas futuras.

[32] Souza MF, Vanderlei LCM, Frias PG (2017). Assessment of the implementation of the Leprosy Control Program
in Camaragibe, Pernambuco State, Brazil. Epidemiol Serv Saude.

[33] Monteiro LD, Lopes LSO, Santos PR, Rodrigues ALM, Bastos WM, Barreto JA (2018). Tendências da hanseníase após implementação de um projeto de
intervenção em uma capital da Região Norte do Brasil,
2002-2016. Cad Saúde Pública.

[34] Silva JSR, Palmeira IP, Sá AMM, Nogueira LMV, Ferreira AMR (2018). Fatores sociodemográficos associados ao grau de incapacidade
física na hanseníase. Rev Cuid.

[35] Souza EA, Heukelbach J, Oliveira MLWDR, Ferreira AF, Sena SA, Raposo MT, Ramos AN (2020). Low performance of operational indicators for leprosy control in
the state of Bahia: spatiotemporal patterns, 2001–2014. Rev Bras Epidemiol.

[36] Ferreira SMB, Ignotti E, Gamba MA (2011). Factors associated to relapse of leprosy in Mato Grosso,
Central-Western Brazil. Rev Saúde Pública.

[37] Feitosa ALM, Dourado FW, Florêncio CMGD (2020). Tendência temporal da hanseníase em uma região de saúde do Ceará,
2001 a 2015. Medicina (Ribeirão Preto).

[38] Bona SH, Silva LOBV, Costa UA, Holanda AON, Campelo V (2015). Recidivas de hanseníase em Centros de Referência de Teresina,
Piauí, 2001-2008. Epidemiol Serv Saúde.

[39] Brasil. Ministério da Saúde (2015). Secretaria de Vigilância em Saúde. Departamento de Vigilância das
Doenças Transmissíveis. Coordenação-Geral de Hanseníase e Doenças em
Eliminação. Nota informativa no 51, de 15 de outubro de 2015. Nota
Informativa sobre recidiva, insuficiência, falência e resistência
medicamentosa na hanseníase [Internet].

[40] Luna IT, Beserra EP, Alves MDS, Pinheiro PNC (2010). Adesão ao tratamento da Hanseníase: dificuldades inerentes aos
portadores. Rev Bras Enferm.

[41] Blok DJ, De Vlas SJ, Richardus JH (2015). Global elimination of leprosy by 2020: are we on
track?. Parasit Vectors.

[42] Pescarini JM, Strina A, Nery JS, Skalinski LM, Andrade KVF, Penna MLF (2018). Socioeconomic risk markers of leprosy in high-burden countries: a
systematic review and meta-analysis. PLoS Negl Trop Dis.

[43] Silvestre MPSA, Lima LNGC (2016). Hanseníase: considerações sobre o desenvolvimento e contribuição
(institucional) de instrumento diagnóstico para vigilância
epidemiológica. Rev Pan-Amaz Saude.

[44] Jesus ILR, Montagner MI, Montagner MA, Alves SMC, Delduque MC (2023). Hanseníase e vulnerabilidade: uma revisão de
escopo. Ciênc Saúde Coletiva.

[45] Batista JFC, Oliveira MR, Pereira DLM, Matos MLSS, Souza IT, Menezes MO (2023). Spatial distribution and temporal trends of AIDS in Brazil and
regions between 2005 and 2020. Rev Bras Epidemiol.

