# Supplementary material for: Quality of healthcare services to reduce leprosy in Brazil: a trend analysis from 2001 to 2020
Source: Rev Bras Epidemiol. 2024 Jul 1;27:e240034. doi: 10.1590/1980-549720240034 (PMC11221484; doi:10.1590/1980-549720240034)
Supplement: Translation

# Translation: Qualidade dos serviços de atenção à saúde para redução da hanseníase no Brasil: análise de tendência de 2001 a 2020

**DOI:** 10.1590/1980-549720240034.2

**Authors:** Gabriel da Silva Mártires, Géssica Liana dos Santos Lima, Danilo Esteves Gomes, Angelina do Carmo Lessa, Celsa da Silva Moura Souza, Eliane Ignotti, Ronilson Ferreira Freitas

**Affiliations:** I Universidade Federal do Amazonas, Faculdade de Medicina – Manaus (AM), Brasil.; II Universidade Federal dos Vales do Jequitinhonha e Mucuri, Programa de Pós-Graduação em Saúde, Sociedade e Ambiente – Diamantina (MG), Brasil.; III Universidade Federal do Amazonas, Programa de Pós-Graduação em Ciências da Saúde – Manaus (AM), Brasil.; IV Universidade do Estado do Mato Grosso, Programa de Pós-Graduação em Ciências Ambientais – Cáceres (MT), Brasil.; V Universidade Federal do Amazonas, Programa de Pós-Graduação em Saúde da Família – Manaus (AM), Brasil.

**Keywords:** Epidemiologia, Distribuição temporal, hanseníase, Indicadores de saúde, Saúde pública

## Abstract

**Objetivo:**

Analisar a tendência temporal dos indicadores de qualidade dos serviços de atenção à saúde para redução da hanseníase no Brasil, no período de 20 anos.

**Métodos:**

Trata-se de um estudo epidemiológico de tendência temporal, cujos dados foram extraídos do Sistema de Informação de Agravos de Notificação (SINAN). Foram construídos indicadores a partir do Manual Técnico-Operacional, que apresenta as Diretrizes para Vigilância, Atenção e Eliminação da Hanseníase como Problema de Saúde Pública do Ministério da Saúde. Para análise de tendência dos indicadores selecionados, utilizou-se o modelo de Prais-Winsten e calculou-se a Taxa de Incremento Anual (TIA).

**Resultados:**

Na série temporal de 20 anos aqui investigada, foram notificados no Brasil 732.959 casos de hanseníase. A tendência mostrou-se estacionária para cura de hanseníase entre os casos novos (β=-0,000; p=0,196; TIA=-0,2); casos de hanseníase em abandono de tratamento entre os casos novos (β=-0,001; p=0,147; TIA=-0,4); contatos examinados de casos novos de hanseníase (β=-0,001; p=0,112; TIA=1,6); casos novos de hanseníase com grau de incapacidade física avaliado no diagnóstico (β=-0,000; p=0,196; TIA=-0,2); e casos curados no ano com grau de incapacidade física avaliada (β=0,002; p=0,265; TIA=0,5); enquanto o indicador casos de recidiva entre os casos notificados no ano (β=0,019; p<0,001; TIA=0,5) apresentou tendência crescente.

**Conclusão:**

Com base na avaliação dos indicadores para mensurar a qualidade dos serviços de atenção à saúde para redução da hanseníase, ficou evidente que o Brasil possui grandes desafios para sua execução plena, sendo necessárias melhorias na qualidade do serviço ofertado à população.

## INTRODUÇÃO

A hanseníase configura-se como um problema de saúde pública devido à sua magnitude e ao alto poder incapacitante, que afeta, sobretudo, populações vulneráveis e marginalizadas em países de média e baixa renda^1^. Mesmo em face de políticas e programas adotados pelo Ministério da Saúde para sua erradicação^2^, a hanseníase continua com elevada prevalência no Brasil^3^ — que, em nível mundial, ocupa o segundo lugar em número de casos —, sendo caracterizada, portanto, como doença negligenciada com potencial de eliminação^3^.

Em 2020 foram notificados 127.396 casos novos da doença no mundo. No continente americano, foram registrados 19.195 (15,1%); destes, 17.979 foram notificados no Brasil, o que corresponde a 93,6% do número de casos novos nas Américas^4^.

Na década de 1980, a Organização Mundial de Saúde (OMS) instituiu a poliquimioterapia (PQT) para o tratamento da hanseníase, visto que esta interrompe a transmissão e previne as deformidades, o que também foi adotado pelo Brasil^5^. Nesse contexto, em 2002 o Ministério da Saúde propôs os indicadores operacionais para avaliação da qualidade de ações e serviços para redução e monitoramento da hanseníase^6^. Em 2016, com a publicação das Diretrizes para Vigilância, Atenção e Eliminação da Hanseníase como Problema de Saúde Pública, os indicadores operacionais foram denominados indicadores para avaliar a qualidade dos serviços de atenção à saúde para redução da hanseníase^7^.

Embora na literatura estejam disponíveis estudos sobre análises de tendências dos indicadores de hanseníase, esses têm focado em contextos locais e/ou regionais, não sendo encontradas publicações com abrangência nacional^8-10^. Adicionalmente, esses estudos avaliaram os indicadores em curtos intervalos temporais^9,11^. Outra lacuna observada é a escassez de estudos de avaliação da qualidade dos serviços de atenção à saúde para redução da hanseníase, visto que as pesquisas, em sua maioria, centraram-se nos indicadores epidemiológicos^11-13^.

Dessa forma, estudos utilizando indicadores para avaliação da qualidade dos serviços de atenção à saúde para redução da hanseníase em nível nacional permitem monitorar o impacto das políticas públicas já implementadas, fornecendo apoio aos gestores em saúde para planejamento, tomada de decisões e execução de programas ou melhorias que abarquem a temática da hanseníase no Brasil. Assim, o presente estudo objetivou analisar a tendência temporal dos indicadores de qualidade dos serviços de atenção à saúde para redução da hanseníase no Brasil, no período de 20 anos.

## MÉTODOS

Estudo epidemiológico de tendência temporal utilizando indicadores de avaliação da qualidade dos serviços de atenção à saúde para redução da hanseníase no Brasil, em série histórica de 2001 a 2020.

Os dados foram obtidos no Sistema de Informação de Agravos de Notificação (SINAN) do Ministério da Saúde, coordenado pelo Departamento de Informática do Sistema Único de Saúde (DATASUS)^14^. Foram selecionadas as notificações com código A-30, pertencentes à categoria “hanseníase”, registradas no período selecionado e que haviam recebido a classificação de acordo com os critérios de Classificação Estatística Internacional de Doenças e Problemas Relacionados à Saúde (Décima Revisão), CID-10^15^.

Para avaliar a qualidade do atendimento dos serviços de saúde para a redução da hanseníase foram utilizados seis indicadores construídos a partir do Manual Técnico-Operacional, que apresenta as Diretrizes para Vigilância, Atenção e Eliminação da Hanseníase como Problema de Saúde Pública do Ministério da Saúde^7^ (Quadro 1).

**Quadro 1:** Indicadores utilizados para avaliar a qualidade dos serviços de atenção à saúde para redução da hanseníase no Brasil^7^.

Indicador	Construção	Utilidade	Parâmetros
Proporção de cura de hanseníase entre os casos novos diagnosticados nos anos das coortes.	Numerador: casos novos de hanseníase residentes em determinado local, diagnosticados nos anos das coortes e curados até 31/12 do ano da avaliação. Denominador: total de casos novos de hanseníase residentes no mesmo local e diagnosticados nos anos das coortes. Fator de multiplicação: 100.	Avaliar a qualidade da atenção e do acompanhamento dos casos novos diagnosticados até a completitude do tratamento.	Bomml: =90%; Regular: =75 a 89,9%; Precário: <75%
Proporção de casos de hanseníase em abandono de tratamento entre os casos novos diagnosticados nos anos das coortes.	Numerador: casos novos de hanseníase diagnosticados nos anos das coortes que abandonaram o tratamento até 31/12 do ano de avaliação. Denominador: total de casos novos diagnosticados nos anos das coortes. Fator de multiplicação: 100.	Avaliar a qualidade da atenção e do acompanhamento dos casos novos diagnosticados até a completitude do tratamento.	Bomml: <10%; Regular: 10 a 24,9%; Precário: =25%.
Proporção de contatos examinados de casos novos de hanseníase diagnosticados nos anos das coortes.	Numerador: nº de contatos dos casos novos de hanseníase examinados por local de residência atual e diagnosticados nos anos das coortes (PB diagnosticados no ano anterior ao ano da avaliação e MB diagnosticados dois anos antes do ano da avaliação). Denominador: total de contatos dos casos novos de hanseníase registrados por local de residência atual e diagnosticados nos anos das coortes (PB diagnosticados no ano anterior ao ano da avaliação e MB diagnosticados dois anos antes do ano da avaliação). Fator de multiplicação: 100.	Mede a capacidade dos serviços em realizar a vigilância de contatos de casos novos de hanseníase, aumentando a detecção oportuna de casos novos.	Bomml: =90,0%; Regular: =75,0 a 89,9%; Precário: <75,0%.
Proporção de casos de recidiva entre os casos notificados no ano.	Número de casos de recidiva de hanseníase notificados/total de casos notificados no ano x 100.	Identificar municípios notificantes de casos de recidiva para monitoramento de falência terapêutica.	Não especifica parâmetro.
Proporção de casos novos de hanseníase com grau de incapacidade física avaliado no diagnóstico.	Numerador: casos novos de hanseníase com o grau de incapacidade física avaliado no diagnóstico, residentes em determinado local e detectados no ano da avaliação. Denominador: casos novos de hanseníase, residentes no mesmo local e diagnosticados no ano da avaliação. Fator de multiplicação: 100.	Medir a qualidade do atendimento nos serviços de saúde.	Bomml: =90%; Regular: =75 a 89,9%; Precário: <75%.
Proporção de casos curados no ano com grau de incapacidade física avaliado entre os casos novos de hanseníase no período das coortes.	Numerador: casos curados no ano com o grau de incapacidade física avaliado por ocasião da cura residentes em determinado local. Denominador: total de casos curados no ano residentes no mesmo local. Fator de multiplicação: 100.	Medir a qualidade do atendimento nos serviços de saúde.	Bomml: =90%; Regular: =75 a 89,9%; Precário: <75%.

O banco de dados foi construído no *software* Microsoft Excel, por meio do qual foram calculadas as proporções. As análises estatísticas para avaliar a tendência temporal e a taxa TIA foram executadas no pacote estatístico Stata (versão 13.0).

Para análise de tendência dos indicadores de avaliação da qualidade do atendimento dos serviços de saúde para redução da hanseníase no Brasil, foi utilizado o teste de regressão linear de Prais-Winsten^16^. Foram consideradas como variáveis dependentes as proporções correspondentes a cada indicador avaliado, enquanto a variável independente correspondeu aos anos da série histórica.

Na análise de tendência, obteve-se o valor de β das proporções de cada indicador avaliado, referente à inclinação da reta. Utilizou-se a taxa de variação para classificar a tendência, sendo que: quando a taxa de variação é positiva, a série temporal é crescente; quando negativa, é decrescente; e é estacionária quando não há diferença significativa entre seu valor e zero. O nível de significância foi dado pela comparação entre o valor de p e o valor dado pela curva normal padrão, com Intervalo de Confiança (IC) de 95%. Considerou-se como significante, para todos os indicadores, aqueles cujo modelo estimado obteve valor de p<0,05^17^.

A estimativa quantitativa da tendência é calculada pela seguinte equação:

$$\text{TIA=}\left({\text{-1+10}}^{\text{β}}\right)\text{*100}$$

Em que:

β corresponde ao coeficiente angular formado na regressão linear.

Para o cálculo do IC das medidas do estudo, utilizou-se a seguinte fórmula:

$$\text{IC95\%=}\left(\text{-1+10}\left(\text{β}\pm \text{r*EP}\right)\right)\text{*100}$$

Em que:

t = valor em que a distribuição *t* de Student apresenta 19 graus de liberdade a um IC95% bicaudal;

EP = erro padrão da estimativa de β, fornecido pela análise de regressão.

Para todos os testes estatísticos realizados, foi adotado o nível de significância de 5%. Assim, foram considerados como significativos valores de p≤0,05.

Seguindo os critérios estabelecidos pela Resolução n° 466/2012^18^ e pela Resolução n° 510/2016^19^ do Conselho Nacional de Saúde para pesquisas envolvendo seres humanos, não necessitam ser registradas nem avaliadas pelo sistema de Comitês de Ética em Pesquisa (CEP)/Comissão Nacional de Ética em Pesquisa (CONEP) pesquisas que utilizem informações de acesso público, nos termos da Lei n° 12.527^20^, de 18 de novembro de 2011.

## RESULTADOS

Na série temporal de 20 anos aqui investigada, foram notificados no Brasil, 732.959 casos de hanseníase. Nesse período, foram registrados 545.610 casos de cura, e a proporção total de cura de hanseníase entre os casos novos diagnosticados nos anos das coortes ao longo da série temporal de 2001 a 2020 foi de 84,49, sendo a menor proporção de cura ocorrida em 2019 e a maior em 2007. Considerando o indicador para avaliação da qualidade do serviço, todos os anos da série — com exceção de 2019, que foi classificado como precário — foram classificados como regular, apontando que, apesar dos esforços para controle e tratamento da hanseníase, estes ainda não são totalmente efetivos no Brasil (Tabela 1).

**Tabela 1:** Qualidade dos serviços de atenção à saúde para redução no Brasil, no período de 2001 a 2020.

Ano	Indicadores					
	Proporção de cura de hanseníase entre os casos novos diagnosticados nos anos das coortes		Proporção de casos de hanseníase em abandono de tratamento entre os casos novos diagnosticados nos anos das coortes		Proporção de contatos examinados de casos novos de hanseníase diagnosticados nos anos das coortes	
	Proporção	Classificação	Proporção	Classificação	Proporção	Classificação
2001	86,97	Regular	6,97	Bom	62,03	Precário
2002	87,23	Regular	5,79	Bom	52,10	Precário
2003	86,05	Regular	5,67	Bom	44,67	Precário
2004	86,56	Regular	5,23	Bom	47,21	Precário
2005	87,16	Regular	4,83	Bom	53,93	Precário
2006	86,26	Regular	5,24	Bom	59,77	Precário
2007	87,47	Regular	5,48	Bom	70,14	Precário
2008	87,18	Regular	5,03	Bom	68,86	Precário
2009	85,36	Regular	4,92	Bom	70,74	Precário
2010	83,72	Regular	4,34	Bom	73,02	Precário
2011	84,44	Regular	4,25	Bom	75,12	Regular
2012	82,77	Regular	4,51	Bom	77,08	Regular
2013	85,16	Regular	4,86	Bom	78,93	Regular
2014	83,76	Regular	5,33	Bom	79,26	Regular
2015	81,68	Regular	6,04	Bom	78,80	Regular
2016	80,74	Regular	6,16	Bom	81,01	Regular
2017	79,09	Regular	6,12	Bom	82,37	Regular
2018	80,70	Regular	6,13	Bom	82,35	Regular
2019	74,68	Precário	7,64	Bom	78,87	Regular
2020	77,07	Regular	6,43	Bom	73,17	Precário

Em relação ao abandono de tratamento, entre os casos novos diagnosticados nos anos das coortes, foram registrados 34.999 casos, cuja proporção de abandono ao longo dos 20 anos da série temporal foi de 5,42%. O ano que registrou o maior número de abandono foi 2019, com 1.971 casos, e o menor número foi registrado em 2011 (1.425). No que se refere ao parâmetro de qualidade do serviço de atenção à saúde para o indicador abandono de tratamento, todos os anos receberam a classificação bom. No período investigado, foram registrados 2.131.711 contatos de casos novos de hanseníase diagnosticados nos anos das coortes, dos quais 1.439.380 foram examinados, apresentando uma proporção de 67,5%. Entre 2001 e 2010, a qualidade do serviço de saúde, no que se refere ao indicador proporção de contatos examinados de casos novos de hanseníase diagnosticados nos anos das coortes, foi classificada como precária, e de 2011 até 2020 foram classificados como regulares, demonstrando, assim, a necessidade de aprimoramento dos serviços de vigilância em saúde (Tabela 1).

Ao longo dos 20 anos, foram registrados 30.873 casos de recidiva, cuja proporção, entre os casos notificados ao longo desses 20 anos, foi de 4,1. A maior proporção de recidiva foi registrada em 2018, e a menor em 2001. Em relação à proporção de casos novos de hanseníase com grau de incapacidade física avaliado no diagnóstico, foi possível observar uma média de 88,9% ao longo de 2001 a 2020, período no qual apenas os anos de 2006, 2009, 2010 e 2011 receberam uma classificação boa — os demais anos, para esse indicador, foram classificados como regular (Tabela 2).

**Tabela 2:** Qualidade dos serviços de atenção à saúde para redução da hanseníase no Brasil, no período de 2001 a 2020.

Ano	Indicadores					
	Proporção de casos de recidiva entre os casos notificados no ano*		Proporção de casos novos de hanseníase com grau de incapacidade física avaliado no diagnóstico		Proporção de casos curados no ano com grau de incapacidade física avaliado entre os casos novos de hanseníase no período das coortes	
	Proporção	Classificação	Proporção	Classificação	Proporção	Classificação
2001	2,7	-	88,29	Regular	62,96	Precário
2002	2,8	-	88,09	Regular	61,33	Precário
2003	2,9	-	88,70	Regular	60,77	Precário
2004	3,1	-	88,66	Regular	60,44	Precário
2005	3,4	-	89,38	Regular	60,67	Precário
2006	3,5	-	90,70	Bom	59,22	Precário
2007	3,5	-	89,15	Regular	70,18	Precário
2008	3,8	-	89,33	Regular	73,36	Precário
2009	3,9	-	90,33	Bom	73,88	Precário
2010	3,9	-	90,69	Bom	73,75	Precário
2011	4,3	-	90,74	Bom	72,25	Precário
2012	4,7	-	89,87	Regular	71,62	Precário
2013	4,6	-	89,47	Regular	71,42	Precário
2014	4,8	-	88,77	Regular	70,30	Precário
2015	5,3	-	88,73	Regular	69,59	Precário
2016	5,6	-	88,64	Regular	69,03	Precário
2017	6,0	-	88,58	Regular	68,45	Precário
2018	6,3	-	88,15	Regular	70,39	Precário
2019	5,7	-	87,29	Regular	68,21	Precário
2020	5,8	-	84,60	Regular	67,81	Precário

* Não especifica parâmetro de classificação.

No que se refere à proporção de casos curados no ano com grau de incapacidade física avaliado entre os casos novos de hanseníase no período das coortes, a proporção média observada entre 2001 e 2019 foi de 67,8%, resultado considerado precário (Tabela 2).

Na análise de tendência temporal dos indicadores de qualidade do atendimento dos serviços de saúde para redução da hanseníase no Brasil, verificou-se tendência estacionária para os seguintes indicadores: proporção de cura de hanseníase entre os casos novos diagnosticados (TIA=-0,2; IC95%=-0,5-0,1); proporção de casos de hanseníase em abandono de tratamento entre os casos novos diagnosticados (TIA=-0,4; IC95%=-1,5-0,2); proporção de contatos examinados de casos novos de hanseníase diagnosticados (TIA=1,6; IC95%=-0,4-3,6); proporção de casos novos de hanseníase com grau de incapacidade física avaliado no diagnóstico (TIA=-0,2; IC95%=-0,5-0,1); e proporção de casos curados no ano com grau de incapacidade física avaliado entre os casos novos de hanseníase no período das coortes (TIA=0,5; IC95%=-0,4-1,5). Por outro lado, o indicador de proporção de casos de recidiva entre os casos notificados no ano (TIA=0,5; IC95%=-0,4-1,5) apresentou tendência crescente (Tabela 3).

**Tabela 3:** Tendência temporal dos indicadores de avaliação da qualidade dos serviços de atenção à saúde para redução da hanseníase no Brasil, no período de 2001 a 2020.

Indicadores	Prais-Winsten					Taxa de Incremento Anual (TIA%)			Tendência
	ß	IC95%		p-valor*	R^2^	TIA%	IC95%		
		Menor	Maior				Menor	Maior	
Proporção de cura de hanseníase entre os casos novos diagnosticados nos anos das coortes	-0,000	-0,002	0,000	0,196	0,999	-0,2	-0,5	0,1	Estacionária
Proporção de casos de hanseníase em abandono de tratamento entre os casos novos diagnosticados nos anos das coortes	-0,001	-0,004	0,000	0,147	0,967	-0,4	-1,0	0,2	Estacionária
Proporção de contatos examinados de casos novos de hanseníase diagnosticados nos anos das coortes	0,006	-0,001	0,015	0,112	0,955	1,6	-0,4	3,6	Estacionária
Proporção de casos de recidiva entre os casos notificados no ano	0,019	0,016	0,021	<0,001	0,916	0,5	-0,4	1,5	Crescente
Proporção de casos novos de hanseníase com grau de incapacidade física avaliado no diagnóstico	-0,000	-0,002	0,000	0,196	0,999	-0,2	-0,5	0,1	Estacionária
Proporção de casos curados no ano com grau de incapacidade física avaliado entre os casos novos de hanseníase no período das coortes	0,002	-0,001	0,006	0,265	0,987	0,5	-0,4	1,5	Estacionária

* IC95%: intervalo de confiança; R^2^: coeficiente de determinação; TIA: taxa de incremento médio anual percentual. *Nível de significância p<0,05.

## DISCUSSÃO

Os achados deste estudo apontam para a precariedade na qualidade da assistência a pessoas diagnosticadas com hanseníase, para sua capacidade em realizar vigilância, bem como para a efetividade das ações para detecção precoce de casos, estando aquém do que é recomendado pela OMS^21^.

A análise de tendência do indicador proporção de cura de hanseníase revelou-se insatisfatória no segmento das pessoas acometidas pela doença até a conclusão do tratamento e obtenção da cura. Estudo realizado no Maranhão entre 2002 e 2015, período próximo ao dos dados aqui apresentados, da mesma forma encontrou tendência estacionária desse indicador, ou seja, sem avanços para o número de casos curados^22^. Machado^23^, em série histórica de 2003 a 2015, analisando clusters de risco para hanseníase no Brasil, também observou tendência estacionária para a proporção de cura na maioria dos clusters, ou seja, em 14 dos 15 analisados. Nesse aspecto, destaca-se a importância do envolvimento dos profissionais de saúde no processo de adesão ao tratamento, bem como a necessidade de programas de educação em saúde para ampliar o conhecimento dos indivíduos com hanseníase sobre a doença, aumentando a proporção de casos curados, e possibilitando, assim, a interrupção da transmissão, bem como casos de incapacidades^23,24^.

Investigou-se a tendência temporal do indicador proporção de casos de hanseníase em abandono de tratamento, que é inversamente proporcional à eficácia do programa de controle da hanseníase^25^, e este apresentou tendência estacionária. Estudo realizado no Brasil no período de 2001 a 2015 relatou um aumento na proporção de abandono do tratamento da hanseníase^26^. Esses achados demonstram a baixa efetividade na estratégia de redução do abandono do tratamento^27^, que poderia resultar em dosagem subterapêutica, levando a resistência aos medicamentos e falha no tratamento^25,28^.

O indicador proporção de contatos examinados de casos novos de hanseníase diagnosticados apresentou tendência estacionária, sugerindo que a capacidade dos serviços em realizar a vigilância de casos novos de hanseníase, bem como sua detecção oportuna, ainda é considerada precária. Mesmo o indicador tendo apresentado, na segunda década, melhoria na classificação, passando de precário a regular, em 2020 a pandemia da COVID-19 possivelmente levou à queda na proporção, e o indicador voltou a ser classificado como precário. Na série histórica apresentada por Souza et al.^29^, com dados do estado da Bahia, a tendência observada entre 2003 e 2014 foi crescente, entretanto, mesmo em face dessa tendência, o indicador também foi classificado como precário. A abordagem do contato é importante para o controle da doença, uma vez que pode gerar aconselhamento e acompanhamento sistemático de longo prazo de indivíduos e famílias expostos ao risco de adoecimento, considerando as características de propagação da doença^29^.

Em relação ao indicador proporção de casos novos de hanseníase com grau de incapacidade física avaliado no diagnóstico, este apresentou tendência estacionária, mantendo padrão regular em todos os anos da série. No estado da Paraíba, esse indicador também apresentou tendência estacionária no período de 2001 a 2014, entretanto, sua classificação se manteve precária em toda a série, não ultrapassando 59%^30^. Já em Minas Gerais, estado pertencente à região com menor ocorrência da doença, embora o indicador tenha sido classificado como bom, ou seja, acima de 90%, no período de 2008 a 2018, sua tendência foi de queda^31^. Dessa forma, é preciso destacar que a tendência e a classificação desse indicador apresentado neste estudo, com dados para o Brasil, escondem a grande variabilidade existente entre regiões do país. Nesse contexto, torna-se relevante monitorar a implementação dos programas de rastreamento ativo de casos de hanseníase, oportunizando o seu controle e a redução da proporção de casos com grau de incapacidade física no momento do diagnóstico^32,33^.

O indicador proporção de casos curados no ano com grau de incapacidade física avaliado entre os casos novos de hanseníase é importante para examinar a eficácia da estratégia de controle da doença, uma vez que o diagnóstico e o tratamento precoces podem reduzir o risco de incapacidade física e, consequentemente, melhorar a qualidade de vida dos pacientes^34^. Observou-se uma tendência estacionária desse indicador, que, durante todos os anos da série investigada, foi classificado como precário. Em Minas Gerais, em estudo realizado no período de 2008 a 2018, esse indicador recebeu uma classificação regular, mas sua tendência decrescente o fez atingir nível precário ao final da série^31^. Esses achados indicam que há desafios persistentes na avaliação e no acompanhamento do paciente pós-cura para, caso haja incapacidade, propiciar assistência para sua reabilitação, de forma a garantir uma assistência integral, conforme preconizada pelo Sistema Único de Saúde (SUS), com base no cuidado e atendimento continuados^11,25,33^.

No presente estudo, o indicador proporção de casos de recidiva entre os casos notificados no ano apresentou uma tendência crescente. Em estudo de série temporal de 2001 a 2014, realizado na Bahia, observou-se tendência crescente para esse indicador, apontando que de 2008 a 2014 houve aumento no número de municípios com notificação de casos de recidiva^35^. Vários são os fatores preditivos de recidiva em pacientes que tenham sido diagnosticados e curados, dentre os quais se destacam falha no tratamento; reinfecção, que se relaciona com as condições de moradia e hábitos de vida; e o modo de organização dos serviços de saúde. Além disso, outro fator que pode influenciar na recidiva é a falha dos profissionais ao distinguir recidivas de quadro reacional após alta^7,36^. Nessa perspectiva, e considerando, ainda, a tendência crescente do indicador, observa-se a necessidade da criação e efetivação de estratégias que proporcionem a diminuição dos determinantes que contribuem para os casos de recidiva^37,38^. É importante que os serviços diferenciem recidiva das situações de reação reversa hansênica, insuficiência terapêutica e falência terapêutica. Os casos que não responderem ao tratamento proposto para os estados reacionais deverão ser encaminhados às unidades de referência para confirmação de recidiva^39^.

Em suma, o Brasil tem um longo caminho a percorrer até que seja possível a erradicação da hanseníase, visto que está entre os cinco países que não alcançaram a meta de controle proposta pela OMS, persistindo com níveis de elevada endemicidade^40^. O número elevado e constante de novos casos é um verdadeiro desafio para a eliminação da hanseníase, pois contribui para que haja novos casos através de contatos domiciliares^41,42^. Ademais, como foi demonstrado neste estudo, os exames de contato, assim como os demais indicadores propostos pela estratégia de enfrentamento da hanseníase, não têm sido alcançados. Além disso, a alta incidência da doença está intimamente ligada a uma determinação social. Dessa maneira, países endêmicos, como o Brasil, deveriam incorporar a erradicação da pobreza como constituinte da sua política de saúde. As ações tomadas dentro do setor da saúde são consideradas insuficientes para atingir a grande gama de necessidades dos despossuídos social e economicamente^7,21,43,44^.

Embora os resultados tenham apontado para a necessidade na melhoria da qualidade do atendimento dos serviços de atenção à saúde para redução da hanseníase, é importante destacar algumas limitações deste estudo. Devido ao desenho epidemiológico empregado, há o impedimento da observação e análise da qualidade da assistência em hanseníase prestada no contexto específico dos municípios brasileiros^9^.

Também se deve levar em conta as limitações advindas do uso de dados secundários do DATASUS, considerando lacunas no preenchimento, além da possibilidade de subnotificação e falhas no fluxo de dados consolidados no sistema. No entanto, seu uso permite o acesso a um registro nacional, que inclui uma população final expressiva, importante para a avaliação epidemiológica de doenças negligenciadas, podendo subsidiar a avaliação por gestores e profissionais, além de auxiliar na tomada de decisão e na revisão de políticas públicas e ações de saúde^17,45^.

Além disso, a análise temporal dos indicadores de efetividade das medidas preventivas e o alcance de suas metas de eliminação da hanseníase no Brasil em uma série de 20 anos permitem avaliar a qualidade dos serviços de atenção à saúde no desenvolvimento de ações de controle, no sentido de gerar evidências que possibilitem a adoção de ações para superar questões operacionais do controle.

Ademais, é importante que sejam realizadas novas análises com estratificação, segundo regiões, para que essas políticas possam ser adaptadas às realidades específicas de cada região do Brasil, dado que a prevalência da doença é heterogênea em todo o país. Devido à grande extensão territorial e à distribuição desigual de recursos, essa adaptação é fundamental para garantir a efetividade das ações de saúde.

Finalmente, considerando o atual quadro da hanseníase no Brasil, é importante que os compromissos propostos pela OMS para a eliminação da doença se configurem em um norte para as ações e políticas implementadas no país, assumindo os desafios de superação do atraso no diagnóstico, tratamento, acompanhamento dos casos e busca ativa dos contatos, com investimentos financeiros que permitam a formação adequada dos profissionais, melhorando a qualidade dos serviços de atenção à saúde para redução da hanseníase no Brasil.
